# Robot-Assisted Minimally Invasive Surgery: First Report from the Caribbean

**DOI:** 10.7759/cureus.18739

**Published:** 2021-10-13

**Authors:** Shamir O Cawich, Tan Arulampalam, Ramdas Senasi, Vijay Naraynsingh

**Affiliations:** 1 Surgery, University of the West Indies, St. Augustine, TTO; 2 School of Medicine, Anglia Ruskin University, Cambridge, GBR; 3 Radiology, Sunderland NHS Foundation Trust, Sunderland, GBR

**Keywords:** mis, invasive, minimal, caribbean, cholecystectomy, robot, laparoscopy

## Abstract

Although advanced minimally invasive surgery and robotic surgery were well accepted in developed countries by the turn of the 21st century, they did not enjoy the same popularity in the Anglophone Caribbean. Advanced minimally invasive surgery only became available in select Caribbean countries from the year 2010. And up to the year 2021, robotic surgery was completely non-existent in the Anglophone Caribbean.

Surgical leaders in the Anglophone Caribbean recognized a need to encourage the introduction of advanced surgical techniques in the region and engaged local and international stakeholders in an attempt to stimulate this development. In the year 2021, through a collaborative effort by a local medical university, a government-funded hospital, and industry partners in the United Kingdom, robot-assisted minimally invasive surgery was successfully introduced to the Caribbean.

We report our experience of introducing robot-assisted minimally invasive surgery in the Eastern Caribbean. By discussing the pitfalls and successes from our experience, we hope that the lessons can be used to guide the introduction of robot-assisted minimally invasive surgery in other resource-poor countries in the Caribbean.​

## Introduction

Seventeen independent countries, with a cumulative population of 7.5 million persons, comprise the Anglophone Caribbean [1]. This includes several low-income countries and some of the poorest countries in the Western Hemisphere [2]. Due to a combination of financial limitations, resource unavailability, and leadership deficiencies, the Caribbean has generally lagged behind the developed world in terms of adopting advanced surgical techniques [3].

As an example, consider the fact that the first laparoscopic cholecystectomy in the Caribbean was performed by Naraynsingh et al. [4] in the year 1992, seven years after it was first described by Eric Muhe in 1985 [5]. The first single incision laparoscopic surgery (SILS) in the Caribbean was performed by Cawich et al. [6] in the year 2009, 16 years after it was first described by Navarra et al. in 1997 [7]. Advanced minimally invasive surgery (MIS) really only gained a firm foothold in the Caribbean in the year 2010 [8-10], almost 20 years after becoming commonplace in the developed world [4].

Similarly, the first robotic procedure was performed by Kwoh et al. [11] in 1988, but robotic surgery was completely non-existent in the Anglophone Caribbean up to the year 2021. Recognizing the need for technical advancement, surgical leaders embarked on a quest to introduce robot-assisted surgery in the Anglophone Caribbean. Our first robot-assisted operation was successfully performed on September 15, 2021, at the Port of Spain General Hospital in Trinidad and Tobago.

We report our experience to demonstrate that robot-assisted MIS is feasible in the Caribbean. We discuss the lessons learned during this exercise. This is important information that can be used to guide the introduction of the technology in other resource-poor countries in the Caribbean and across the globe.

## Case presentation

A 25-year-old woman with no medical illnesses presented to the hospital complaining of right upper quadrant pain and vomiting. The abdomen was soft and non-tender. An elective abdominal ultrasound confirmed the presence of cholelithiasis. The common bile duct was normal in caliber and liver function tests were within normal limits. She was scheduled for elective cholecystectomy.

After induction of general anesthesia, an open technique was used to insert a 12 mm laparoscopic port at the umbilicus. Two 5 mm working ports were inserted in the right upper quadrant of the abdomen for surgeon dissection. Only three ports were used to perform the cholecystectomy. The choice of port placement was made to provide maximal surgeon ergonomics and no modification of our usual port placement was required to facilitate the use of the robotic arm.

**Figure 1:**
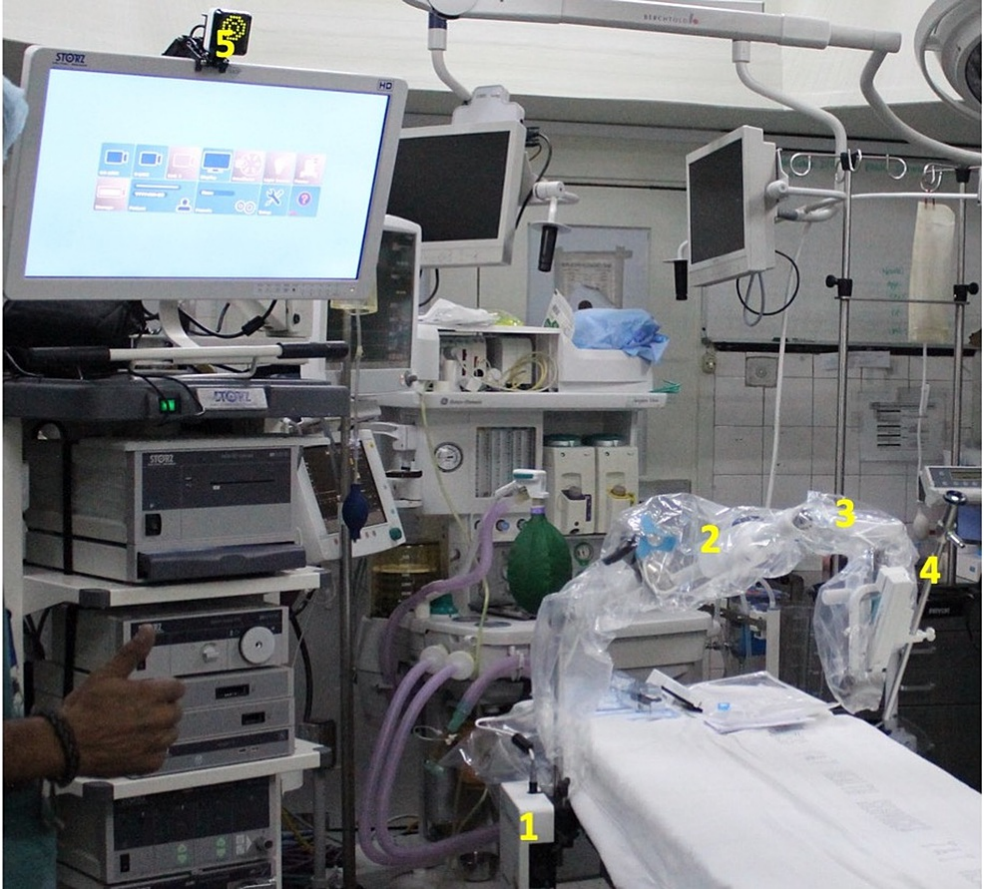
Placement of the Freehand® robotic arm The Freehand® robotic arm is seen in place, fixated to the operating table rail. The robotic arm components are visible: control box (1), robotic mechanical arm (2), robot control module (3), conventional 10 mm laparoscope (4), and infrared sensor (5).

The Freehand® robotic arm was secured to the operating table and used to fixate a 10 mm zero-degree laparoscope that was introduced into the umbilical port (Figure 1). The Freehand® robotic arm was completely set up in approximately two minutes and did not consume a significant amount of operating time in this case.

**Figure 2:**
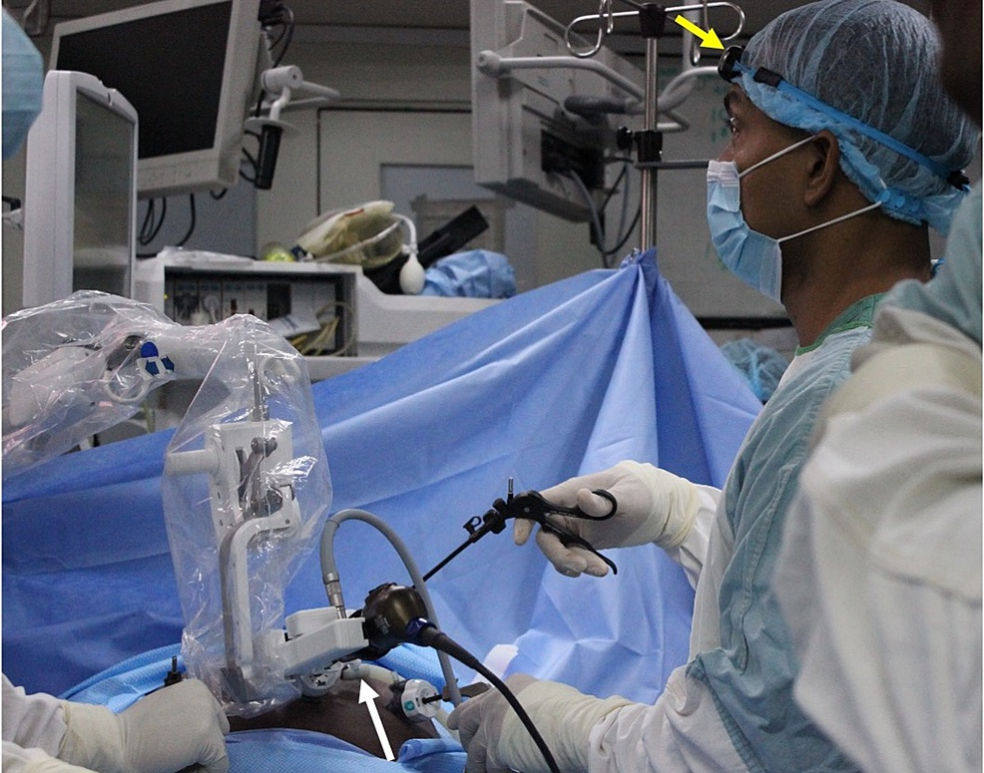
Intraoperative view of the robotic arm in use The operating surgeon controls the robotic arm (white arrow) using head movements relayed by an infrared transmitter (yellow arrow) worn on a headband.

**Figure 3:**
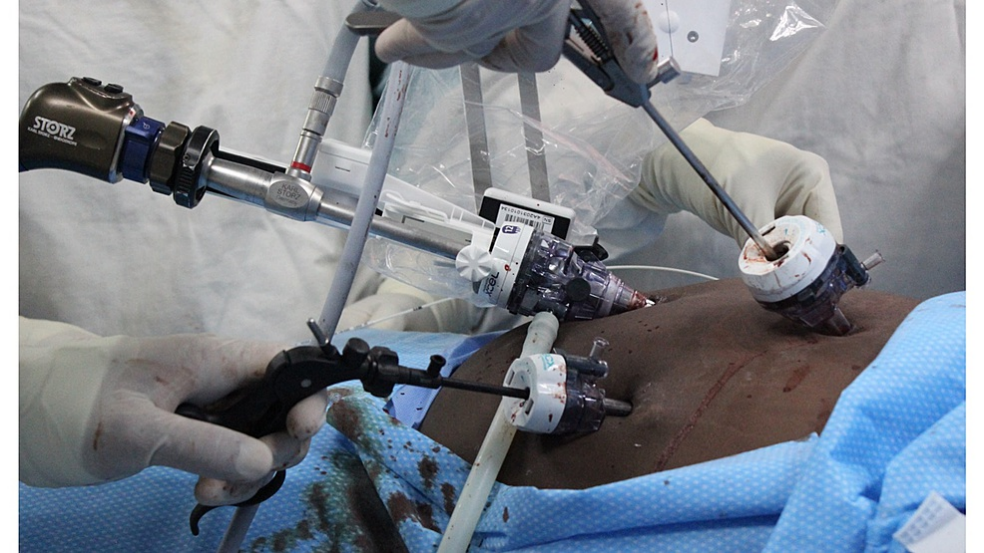
Detailed view of the operating field Detailed view of the operating field showing the setup of the robotic arm clasping and controlling the laparoscope while the surgeon uses conventional instruments in working ports.

**Figure 4:**
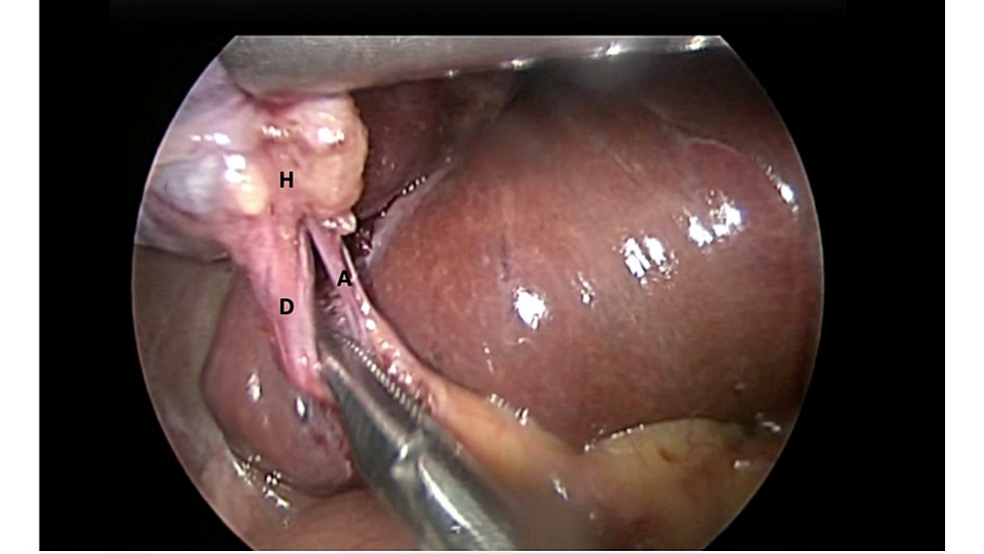
Intraoperative view from the robotic arm Intracorporeal view of the critical view of safety, with the cystic duct (D), cystic artery (A), and Hartmann’s pouch (H) clearly visible.

The surgeon controlled the Freehand® robotic arm using an infrared communication device fixated on a headpiece (Figure 2). This allowed the surgeon full control of the laparoscope using the robotic arm (Figure 3). The working instruments were controlled by the surgeon and used to demonstrate Strasberg’s critical view prior to fixation of the cystic structures (Figure 4). The operation was completed in 30 minutes. During this time, camera cleaning was required on two occasions. To facilitate this, the scope was easily disconnected from the robotic arm, cleaned in the usual fashion, and reconnected to the robotic arm. The procedure to clean the scope consumed less than one minute of operating time.

At the end of the operation, the robot was removed and the specimen was extracted using the umbilical port. There were no complications detected. The patient was discharged from the hospital within 24 hours and recovered uneventfully.

## Discussion

Although it enjoyed widespread popularity across the globe [12], robotic surgery was non-existent in the English-speaking Caribbean prior to the year 2021. There are many reasons for the lag in the adoption of robotic surgery. Consider the fact that many of the countries in the Caribbean are developing countries that fall within the low-to-middle income categories of the World Health Organization [13]. These resource-poor countries would find the cost to acquire commercially available robotic surgery systems prohibitive.

In addition, many of the countries have relatively small populations. Only four Caribbean countries have populations of greater than 250,000 persons [1], and some have populations as small as 5,000 persons [13]. Many Caribbean Governments cannot justify the procurement of a robotic system when there may not be sufficient cases within a small population. Finally, the same obstacles that open surgeons mounted against laparoscopic surgery seem to have come full circle, with laparoscopic surgeons in the Caribbean now being fierce opponents to the introduction of robotic surgery [14-15].

The first robot-assisted laparoscopic operation in the English-speaking Caribbean was performed on September 15, 2021, at the Port of Spain General Hospital in Trinidad & Tobago by Cawich et al. This is 33 years after Kwoh et a. [11] performed the first robotic procedure in 1988 using the PUMA 560 robotic system for neurosurgical biopsies. And it is 30 years after Davies et al. [16] used the PUMA 560 robotic system to perform trans-urethral resections of the prostate in 1991.

Shortly after these surgeons pioneered robotic surgery, we saw rapid advancements in technology and equipment, predominantly driven by the United States military’s pursuit of telepresence in order to reduce mortality in battlefield operations [17]. This culminated in the development of the da Vinci® system from Intuitive Surgical Inc (Sunnyvale, California, USA), which dominated the robotic surgery landscape over the subsequent two decades [17]. While there are numerous and well-documented advantages associated with the da Vinci® system, the purchase price is prohibitive to many developing nations. In addition, there are no regional distributors from Intuitive Surgical to service the Caribbean.

There are cheaper alternatives that have been developed that are also advantageous. The AESOP (Automated Endoscopic System for Optimal Positioning) robotic platform utilized voice recognition to control laparoscopic camera positioning [17]. Modifications of this system led to the development of the ZEUS operating system. The EndoAssist used infrared communication between a robotic arm and a headset worn by the operating surgeon [17]. The Freehand® system utilizes similar radiofrequency technology to allow the surgeon to control the robotic arm handling the laparoscope.

In our experience, the Freehand® system is a good intermediary that costs significantly less than the da Vinci® system but provides some advantages over traditional laparoscopy. First, the surgeon controls the visual field, eliminating human error by the camera person. This becomes especially important for long and technically complex operations such as liver or pancreatic surgery. Second, head movements to control the robot are similar to the direction the surgeon would look in order to view the operative field, making control of the robot quite intuitive and not detracting from the surgeon’s control of the operating instruments.

Furthermore, in the pandemic era, when operating room staff is deliberately skeletonized to prevent potential viral spread, the Freehand® system has an obvious advantage to reduce exposed staff numbers. Additionally, the surgical discipline is not a particularly relevant specialty when it comes to the pandemic response. Therefore, many of the junior surgical staff have been re-deployed to work on coronavirus disease (COVID) response teams and/or vaccination drives. In this setting, the Freehand® system mitigates surgical staff shortage while reducing the operating team exposure.

With appropriate training, we believe this technology can be introduced to other developing countries within the region. However, before embarking on the operation, the primary surgeon received extensive training in a dry lab and intraoperative mentoring from experts familiar with the technology. With these precautions, the surgeon was able to complete a robot-assisted laparoscopic cholecystectomy with morbidity and mortality comparable to that reported from conventional laparoscopic procedures [2,8-10].

The fact that this procedure was completed successfully by advanced laparoscopic surgeons trained in Caribbean centers is testimony to the maturation of MIS training in Caribbean institutions. This report should also motivate MIS surgeons in the region to support the development of robotics within the Caribbean. We advocate for Freehand® technology to be used by trained and experienced laparoscopic surgeons who are familiar with the proposed MIS procedure.

## Conclusions

Despite operating in an under-funded environment, robot-assisted MIS is feasible in the Caribbean. There are many commercial platforms available, but the Freehand® system appears to be a good intermediary that balances cost while (1) eliminating human error to maintain vision, (2) using intuitive motions that easily control the robot, (3) reducing surgeon crowding at the operating table, and (4) allowing surgical services to effectively function when human resources are limited. Before using the technology, we advocate surgeons should undergo a period of dry lab training as well as intraoperative mentoring from surgeons familiar with the technology.
